# C. H. Collins

**Published:** 1910-03

**Authors:** 


### C. H. COLLINS, M.R.C.S., L.S.A.,
Chew Magna.

Charles Howell Collins died at Chew Magna on December 22nd, 1909, in his 87th year.

Born at Devizes in 1822, he came of an old Welsh family. His father, Robert Benjamin Collins, also a medical man, began practice in Chew Magna in 1824, and in that village the subject of this notice spent his long life. In his day there were few better known medical men in the neighbourhood of Bristol. His fine presence, courtly manners, thorough knowledge of his profession, and genuine kindness, inspired a confidence and love amongst his numerous patients rarely exceeded, and he had the faculty of retaining friendships once made.

He studied medicine at the Bristol School and St. Bartholomew's Hospital, qualifying M.R.C.S., L.S.A., in 1846, and by the death of his father in the same year had to take over the practice at Chew Magna.

He was Medical Officer of the Clutton Union, Surgeon to the Odd Fellows and other clubs, and at one time or another held all the appointments open to him.

He was President of the Bath and Bristol Branch of the British Medical Association as long ago as 1869, took a keen interest in professional matters to the very last, and kept himself well abreast of the times.

He was also much interested in all matters that he thought were for the good of the district, and took an active part in church and educational affairs. He was a thorough sportsman and supporter of all agricultural interests.

Maintaining his intellectual activity to the last, his stories of medical life and practice in Bristol were most interesting. He thought his successor had a much easier time of it getting over the district in a motor than he had in the saddle.

His funeral at Chew Magna on December 27th was largely attended, and "the old doctor" will be missed and mourned for many a day by numbers who were proud to call him friend.

